# Supplementary material for: Stress drives plasticity in leaf ageing transcriptional dynamics in Arabidopsis thaliana
Source: Nat Plants. 2026 Mar 19;12(4):780–90. doi: 10.1038/s41477-026-02254-3 (PMC13106033; doi:10.1038/s41477-026-02254-3)
Supplement: Supplementary file 1 — Reporting Summary [file 41477_2026_2254_MOESM1_ESM.pdf]

nature portfolio

Corresponding author(s): Joseph Swift, Joseph Ecker

Last updated by author(s): 1/2/2026

Reporting Summary

Nature Portfolio wishes to improve the reproducibility of the work that we publish. This form provides structure for consistency and transparency in reporting. For further information on Nature Portfolio policies, see our Editorial Policies and the Editorial Policy Checklist.

Statistics

For all statistical analyses, confirm that the following items are present in the figure legend, table legend, main text, or Methods section.

n/a	Confirmed	
☐	☒	The exact sample size (*n*) for each experimental group/condition, given as a discrete number and unit of measurement
☐	☒	A statement on whether measurements were taken from distinct samples or whether the same sample was measured repeatedly
☐	☒	The statistical test(s) used AND whether they are one- or two-sided<br>*Only common tests should be described solely by name; describe more complex techniques in the Methods section.*
☐	☒	A description of all covariates tested
☐	☒	A description of any assumptions or corrections, such as tests of normality and adjustment for multiple comparisons
☐	☒	A full description of the statistical parameters including central tendency (e.g. means) or other basic estimates (e.g. regression coefficient) AND variation (e.g. standard deviation) or associated estimates of uncertainty (e.g. confidence intervals)
☐	☒	For null hypothesis testing, the test statistic (e.g. *F*, *t*, *r*) with confidence intervals, effect sizes, degrees of freedom and *P* value noted<br>*Give P values as exact values whenever suitable.*
☒	☐	For Bayesian analysis, information on the choice of priors and Markov chain Monte Carlo settings
☒	☐	For hierarchical and complex designs, identification of the appropriate level for tests and full reporting of outcomes
☐	☒	Estimates of effect sizes (e.g. Cohen's *d*, Pearson's *r*), indicating how they were calculated

Our web collection on statistics for biologists contains articles on many of the points above.

Software and code

Policy information about availability of computer code

Data collection	no software was used for data collection
Data analysis	The following R packages were used: Seurat (v5.2.1), DESeq2 (v1.46.0), ggplot2 (v4.0.0), EDASeq (v2.40.0), dplyr (v1.1.4), data.table (v1.17.0), plyranges (v1.26.0), cowplot (v1.2.0), viridis (v0.6.5), VennDiagram (v1.7.3), rtracklayer (v1.66.0), plyr (v1.8.9), reshape2 (v1.4.4), gridExtra (v2.3), gplots (v3.2.0), plotly (v4.10.4), splines (v4.4.3), igraph (v2.1.4), Matrix (v1.7.3), devtools (v2.4.5), and stringr (v1.5.1).<br>R code is deposited on GitHub at https://github.com/joey1463/Leaf-by-Leaf-Drought-Atlas.git

For manuscripts utilizing custom algorithms or software that are central to the research but not yet described in published literature, software must be made available to editors and reviewers. We strongly encourage code deposition in a community repository (e.g. GitHub). See the Nature Portfolio guidelines for submitting code & software for further information.

Data

Policy information about availability of data

All manuscripts must include a data availability statement. This statement should provide the following information, where applicable:

- Accession codes, unique identifiers, or web links for publicly available datasets
- A description of any restrictions on data availability
- For clinical datasets or third party data, please ensure that the statement adheres to our policy

Single-cell RNA-seq data are publicly available through GEO (GSE290214). Nuclei data are hosted at the Klarman Cell Observatory at: https://singlecell.broadinstitute.org/single_cell/study/SCP2703). Microscopy data reported in this paper will be shared by the lead contact upon request.

Research involving human participants, their data, or biological material

Policy information about studies with human participants or human data. See also policy information about sex, gender (identity/presentation), and sexual orientation and race, ethnicity and racism.

Reporting on sex and gender	not applicable
Reporting on race, ethnicity, or other socially relevant groupings	not applicable
Population characteristics	not applicable
Recruitment	not applicable
Ethics oversight	not applicable

Note that full information on the approval of the study protocol must also be provided in the manuscript.

Field-specific reporting

Please select the one below that is the best fit for your research. If you are not sure, read the appropriate sections before making your selection.

☒ Life sciences ☐ Behavioural & social sciences ☐ Ecological, evolutionary & environmental sciences

For a reference copy of the document with all sections, see nature.com/documents/nr-reporting-summary-flat.pdf

Life sciences study design

All studies must disclose on these points even when the disclosure is negative.

Sample size	For our individual leaf drought time course we chose to harvest and analyze 6 plants per time point (9 time points total). We selected our time points through previous experimental evidence based off bulk transcriptomics that indicated this was the sufficient number of samples to detect gene expression differences.<br>For our individual seedling time course experiment we chose to harvest and analyze 6 plants per time point, per treatment condition (11 time points total). We selected our time points through previous experimental evidence based off bulk transcriptomics that indicated this was the sufficient number of samples to detect gene expression differences.
Data exclusions	No significant data was excluded from our study. Only genes with low read counts, or nuclei with low UMIs, were removed during normal processing of single-nuclei sequencing data.
Replication	Single nuclei sequencing data presented in this study was replicated in the following ways:<br>- For our individual leaf transcriptome sequencing experiment, we assayed 3 individual plants. We repeated this experiment in a second true biological replicate within a separate time course (6 individual plants total), which yielded comparable results.<br>- For our individual seedling transcriptome sequencing experiment, we assayed 6 - 12 individual plants selected across 3 or more separate agar plates.<br>For our FRO6 transgenic growth assays, we replicated each test on 2 or greater times - all attempts at replication were successful.
Randomization	Plants (both whole rosettes and seedlings) were randomized every second or third day within the growth chamber.
Blinding	Blinding was not possible because experimental groups were defined by visible phenotypic traits and treatment conditions that could not be concealed during data collection

Reporting for specific materials, systems and methods

We require information from authors about some types of materials, experimental systems and methods used in many studies. Here, indicate whether each material, system or method listed is relevant to your study. If you are not sure if a list item applies to your research, read the appropriate section before selecting a response.

Materials & experimental systems

n/a	Involved in the study	
☒	☐	Antibodies
☒	☐	Eukaryotic cell lines
☒	☐	Palaeontology and archaeology
☒	☐	Animals and other organisms
☒	☐	Clinical data
☒	☐	Dual use research of concern
☐	☒	Plants

Methods

n/a	Involved in the study	
☒	☐	ChIP-seq
☒	☐	Flow cytometry
☒	☐	MRI-based neuroimaging

Dual use research of concern

Policy information about dual use research of concern

Hazards

Could the accidental, deliberate or reckless misuse of agents or technologies generated in the work, or the application of information presented in the manuscript, pose a threat to:

No	Yes	
☒	☐	Public health
☒	☐	National security
☒	☐	Crops and/or livestock
☒	☐	Ecosystems
☒	☐	Any other significant area

Experiments of concern

Does the work involve any of these experiments of concern:

No	Yes	
☒	☐	Demonstrate how to render a vaccine ineffective
☒	☐	Confer resistance to therapeutically useful antibiotics or antiviral agents
☒	☐	Enhance the virulence of a pathogen or render a nonpathogen virulent
☒	☐	Increase transmissibility of a pathogen
☒	☐	Alter the host range of a pathogen
☒	☐	Enable evasion of diagnostic/detection modalities
☒	☐	Enable the weaponization of a biological agent or toxin
☒	☐	Any other potentially harmful combination of experiments and agents

Plants

Seed stocks	We used wild type Arabidopsis, as well as generated 2 transgenic lines overexpression the FRO6 gene.
Novel plant genotypes	A 4198 bp genomic fragment from the FRO6 locus, which contains the FRO6 coding region, 886 bp upstream of ATG, and 211 bp of 3' UTR, was used to generate FRO6p::FRO6:GFP transgenic line. GFP was translationally fused to the C-terminus of FRO6 before the stop codon. For the TSSp::FRO6:GFP transgenic line, a 1643 bp fragment upstream of TSS start codon was used to replace the FRO6 promoter. The same TSSp fragment was used in generating the TSSp::GUS transcriptional fusion. All transgenes were cloned into the binary vector pMX202 and stably transformed into Arabidopsis Col-0 background.
Authentication	The presence of the transgene was confirmed by growth on selection media (kanamycin), then verified by visualizing GFP expression. 20 positive lines met this criteria. Single inserts were chosen from these lines, and then homozygosed.
